# A novel deep autoencoder based survival analysis approach for microarray dataset

**DOI:** 10.7717/peerj-cs.492

**Published:** 2021-04-21

**Authors:** Hanaa Torkey, Mostafa Atlam, Nawal El-Fishawy, Hanaa Salem

**Affiliations:** 1 Computer Science & Engineering Department, Faculty of Electronic Engineering, Menoufia University, Menouf, Egypt; 2 Faculty of Engineering, Delta University for Science and Technology, Gamasa, Egypt

**Keywords:** Survival analysis, Deep learning, Autoencoder, Breast cancer, Cox regression, Graphical processing unit, RNAseq data

## Abstract

**Background:**

Breast cancer is one of the major causes of mortality globally. Therefore, different Machine Learning (ML) techniques were deployed for computing survival and diagnosis. Survival analysis methods are used to compute survival probability and the most important factors affecting that probability. Most survival analysis methods are used to deal with clinical features (up to hundreds), hence applying survival analysis methods like cox regression on RNAseq microarray data with many features (up to thousands) is considered a major challenge.

**Methods:**

In this paper, a novel approach applying autoencoder to reduce the number of features is proposed. Our approach works on features reconstruction, and removal of noise within the data and features with zero variance across the samples, which facilitates extraction of features with the highest variances (across the samples) that most influence the survival probabilities. Then, it estimates the survival probability for each patient by applying random survival forests and cox regression. Applying the autoencoder on thousands of features takes a long time, thus our model is applied to the Graphical Processing Unit (GPU) in order to speed up the process. Finally, the model is evaluated and compared with the existing models on three different datasets in terms of run time, concordance index, and calibration curve, and the most related genes to survival are discovered. Finally, the biological pathways and GO molecular functions are analyzed for these significant genes.

**Results:**

We fine-tuned our autoencoder model on RNA-seq data of three datasets to train the weights in our survival prediction model, then using different samples in each dataset for testing the model. The results show that the proposed AutoCox and AutoRandom algorithms based on our feature selection autoencoder approach have better concordance index results comparing the most recent deep learning approaches when applied to each dataset. Each gene resulting from our autoencoder model weight is computed. The weights show the degree of effect for each gene upon the survival probability. For instance, four of the most survival-related experimentally validated genes are on the top of our discovered genes weights list, including PTPRG, MYST1, BG683264, and AK094562 for the breast cancer gene expression dataset. Our approach improves the survival analysis in terms of speeding up the process, enhancing the prediction accuracy, and reducing the error rate in the estimated survival probability.

## Introduction

Breast cancer (Gewaifel et al., 2019) is one of the deadliest diseases all over the world. Breast cancer transpires among women around the world as one of the major causes of deathly diseases. However, there is evidence that early diagnosis and care will increase the survival rate of patients with breast cancer (Salem, Attiya & El-Fishawy, 2016). In Egypt, breast cancer is almost 18.9% of all cancer cases (32.04% in women and 2.2% in men). In the United States, breast cancer is the most common cancer in women after skin cancer. The appearances of DNA microarrays empowered the simultaneous monitoring of expression levels of thousands of genes and have led to rise of using machine learning techniques to build classification and survival models. Many methods for accurately predicting the importance of each feature and patient survival probability were developed based on symptom data and basic clinical parameters (Grqnnesby & Borgan, 1996).

Survival analysis is a model for time until a certain event. Time-to-event data would face many challenges like censoring, convergence problems, high-dimensionality, and temporal dependencies (Fadnavis, 2019). Cox Proportional Hazards Model (Cox) (Cox, 1975) and random survival forests are considered the most common methods used in survival analysis. Survival analysis methods like cox regression are built to deal with clinical features (up to hundreds), so dealing with features up to thousands would be considered as a problem that can result in a computer crash (Wen & Zhang, 2018). There are some other survival analysis methods like random survival methods that can deal with a large number of features, but this would affect the survival accuracy. Survival analysis approaches (Cox, 1972) can have three categories, parametric, non-parametric and semi-parametric models. The parametric approach isn’t suitable for normal distribution. The semi-parametric approach can be considered as a combination of parametric and non-parametric approaches. In a semi-parametric approach, the regression parameters could be known, but the distribution of the survival time would be still unknown (Wang & Li, 2017b). Non-parametric approaches are suitable when the theoretical distribution of the data is not known.

When survival analysis method is applied on RNAseq data with features up to thousands to predict survival probability and significant features (genes) it face a computational complexity problem. In order to reduce features and solve this problem of handling thousands of features; additional methods could be applied like dimensionality reduction and feature selection methods.

Principal Component Analysis (PCA) is one of the most popular technique for dimensionality reduction. PCA is a statistical technique that transforms the original numeric dimensions of size N features into a new set of *n* dimensions called principal components. In this transformation, the first principal component has the largest possible variance and each succeeding principal component has the highest possible variance under the constraint that it is uncorrelated with the preceding components. Then the method Keeps only the first *m* < *N* components to reduces the feature dimensionality. It is known that PCA transformation is sensitive to the scaling of the original feature values and therefore the feature values needed to be normalized. Other techniques, like Linear Discriminant Analysis (LDA), Non-negative Matrix Factorization (NMF), and Singular Value Decomposition (SVD) are also often used for dimensionality reduction (Van Der Maaten, Postma & den Herik, 2009).

Dimensionality reduction methods (Torkey et al., 2020) transform a high-dimensional space into a low-dimensional space of data, producing a new representation of features. The new representation of features is considered a problem for survival analysis methods because it eliminates the property of predicting the importance of original features (genes) as it produces a new representation of features. Feature selection methods (Chandrashekar & Sahin, 2014) select features based on the target variable, which can be difficult here because there is no specific target variable in survival analysis as it is based on event and duration variables to build the survival model. Recent researches prove that Deep Learning (DL) is the best way to deal with feature selection in survival analysis models (Zahangir et al., 2019). The performance of DL models is better than traditional ML algorithms. These models are evaluated in terms of concordance index, which findings have highlighted the relationships between patient characteristics and survival analysis (Huang et al., 2020). One of the first survival analysis approach based on deep learning to compute survival times is proposed and applied on a breast cancer dataset (Lee et al., 2018) is called DeepHit. DeepHit makes no assumptions about the underlying stochastic process. Artificial neural networks are deployed in survival analysis applied on two breast cancer datasets used in DeepHit were generated using the Xcyt image analysis program (Chi, Street & Wolberg, 2007). Where predicting recurrence probability and separating patients with good (more than five years) and bad (less than five years).

Another DL model was applied to the clinical dataset for cancer proposing a system called DeepSurv proposed by Kim et al. (2019) (Hinton & Salakhutdinov, 2006). DeepSurv approach enhances the performance compared with random survival forest and the Cox proportional hazard model. Where, DeepSurv is used to reconstruct features and remove noise to solve the problem of high collinearity and convergence. After applying deep learning and removing noise, the zero variance features was removed as they will not affect the model. DeepSurv performed best when compared with RSF and the CPH models with c-index on testing sets up to 0.781. It showed a relatively steadier upward trend, while RSF and CPH models showed decreases when statistically insignificant features were added.

Hao et al. (2019) integrate multiple types of data (like, microarray data and clinical data for the same patients) in a deep learning model, named Cox-PASNet. Co-PASNet is a sparse pathway-based deep neural network, which consists of five layers; genes input layer, pathway layer, some hidden layers, clinical date layer, and finally Cox layer. The gene layer and clinical data layer are merged at the last hidden layer in order to estimate the prognostic index as an input for cox regression. (Chi, Street & Wolberg, 2007; Liou et al., 2014) ANNs to the survival analysis problem of a BC dataset. The advantage of using ANNs was that it could successfully predict recurrence probability and separate patients with good and bad prognoses. The disadvantage was that the results were unclear when separating into subgroups.

Ranganath et al. (2016) introduced another deep survival analysis model using hierarchical generative approach in the context of the Electronic Health Record (EHR). The introduced model handled heterogeneous data types that occur in the HER. The model was compared to the clinically validated Framingham risk score. From these studies deep survival analysis was discovered superior in stratifying patients according to their risk (Ching, Zhu & Garmire, 2018; Wang et al., 2014). Table 1 displays a comparison among the recent survival analysis model that use deep learning on expression datasets with many features.

**Table 1:** Comparison analysis for the existing methods in terms of concordance index.

**References**	**Algorithm**	**Dataset**	Concordance
Lee et al. (2018); Chi, Street & Wolberg (2007)	cause-specific version of the Cox Proportional Hazards Model (cs-Cox)	METABRIC Breast Cancer Dataset	0.639 Range: (0.633–0.645)
	DeepHit		0.691 Range: (0.679–0.703)
	DeepHit (*α* = 0)		0.646 Range: (0.634–0.658)
	Cox		0.648 Range: (0.634–0.662)
Hao et al. (2019)	Cox-PASNet	Integrated microarray gene expression and clinical data for Ovarian serous cystadenocarcinoma (OV) cancers	0.6347 Range (0.5975 –0.679)
Ranganath et al. (2016)	Deep Survival Analysis	Heart Disease	0.7311 With Risk Score = 50
Chi, Street & Wolberg (2007)	ANN	Wisconsin Prognostic Breast Cancer (WPBC)	The error bars are 95% confidence intervals from the predicted survival function. ANNs can successfully predict recurrence probability and separate patients with good (more than five years) and bad (less than five years) prognoses. Results are not as clear when the separation is done within subgroups such as lymph node positive or negative.
Kim et al. (2019)	DeepSurv	Medical records of oral squamous cell carcinoma patients	Training Concordance: 0.810 Testing Concordance: 0.781
	RSF		Training Concordance: 0.770 Testing Concordance: 0.764
	CPH model		Training Concordance: 0.756 Testing Concordance: 0.694

The main objectives in this paper are to define the main features affecting the survival probability for breast cancer with the aid of the most suitable machine learning algorithm. This information can help doctors in making the right decision about each patient’s case according to the available treatment and medical instruments. The current survival analysis methods are lacking when applying to datasets with thousands of features, like in microarray datasets Sakurada & Yairi (2014). Our work focuses on developing deep learning model to detect the most significant features, from gene expression datasets that could be employed in enhancing the survival probability prediction, and biomarker discovery.

The proposed model developed an Autoencoder (AE) deep learning model (Liou et al., 2014) for feature selection and feature importance discovery. Then, it utilizes Cox regression and random survival forest algorithms for survival probability estimation. The autoencoder deep neural network is used to reconstruct features, remove noise and leave features with variance by which the output can be affected .Then survival analysis method is applied to the selected and reconstructed features to predict the survival probability. Our model also estimates the importance of each feature by computing *P*-value for each feature. Applying an autoencoder upon features that are up to nearly tens thousand would take long time, so our autoencoder model is applied on GPU to speed up the model training. The proposed model is finally evaluated on three datasets in terms of run time, concordance index and calibration curve, and the *p*-value for the prediction probability. The open source is provided for our model implementation using Python at https://github.com/Mostafa-Samy-Atlam/Autoencoder-with-survival-analysis-methods.

The main contributions of this research could be summarized as the following:

• Developing an autoencoder deep neural network to feature selection, reconstruct feature and remove zero variance features removal. This autoencoder is applied on GPU for speeding up the process.
• Finding the survival probability for each patient by applying cox regression and random survival analysis on the generated features from the proposed autoencoder model.
• Estimating the impact of each feature on survival probability by computing *p*-value for each feature can be used as an indication for the importance of each feature on survival probability, which enables the model to discover gene biomarkers for breast cancer survival.

This paper is organized as follows: The proposed survival analysis model design and implementation are introduced in ‘Proposed Survival Analysis Model’. Then ‘Results’ introduces the results and discussion. Finally, the conclusion and future direction are discussed in ‘Conclusion’.

## Proposed Survival Analysis Model

**Figure 1:**
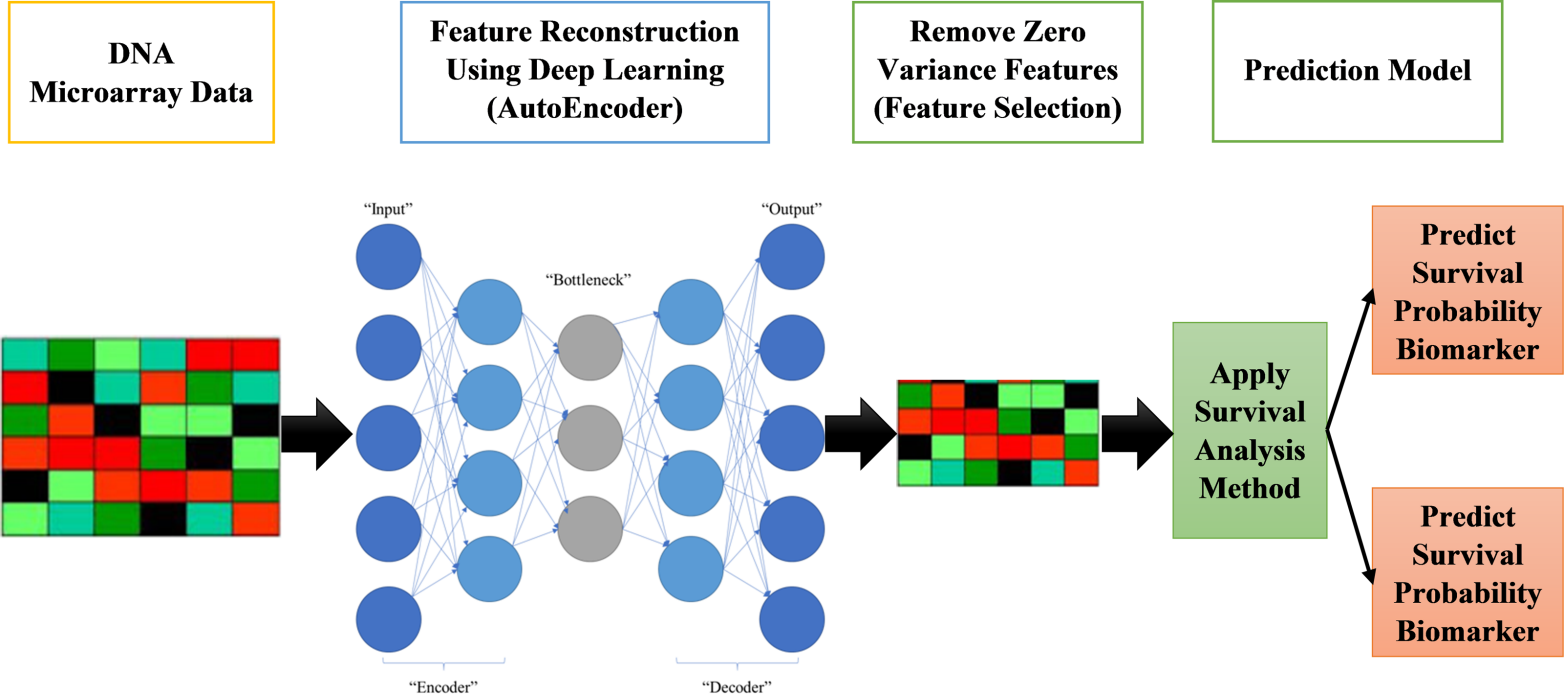
Proposed survival analysis approach.

The proposed systems in this paper are called AutoCox and AutoRandom. These two models are used to define significant genes (features) that differentiate between sever and death cases and predict survival probability. As shown in Fig. 1, the proposed model is made up of four major stages:

• Reading datasets, solving categorical data problems, and handling missing data are all done in the first stage of data preprocessing.
• The second stage is the training model, which includes applying an autoencoder deep neural network for feature selection by feature reconstruction and noise removal.
• Applying Cox regression or Random Survival Forests for survival analysis calculation. The autoencoder deep neural network is applied on GPU for speeding up the process.

Finally, survival prediction (whether alive or dead) and discovering the most significant features/genes, the features that have a major role in affecting the survival probability estimation.

### Dataset preprocessing

The proposed system starts with reading dataset then data preprocessing in order to make the data ready for applying the training model. It starts with handling missing data. In our model implementation two processing steps are performed on the datasets. First, each missing value is replaced with the mean of the values in the samples of class it belongs to. Second, the values in all samples are normalized.

### Autoencoder deep neural network model

**Figure 2:**
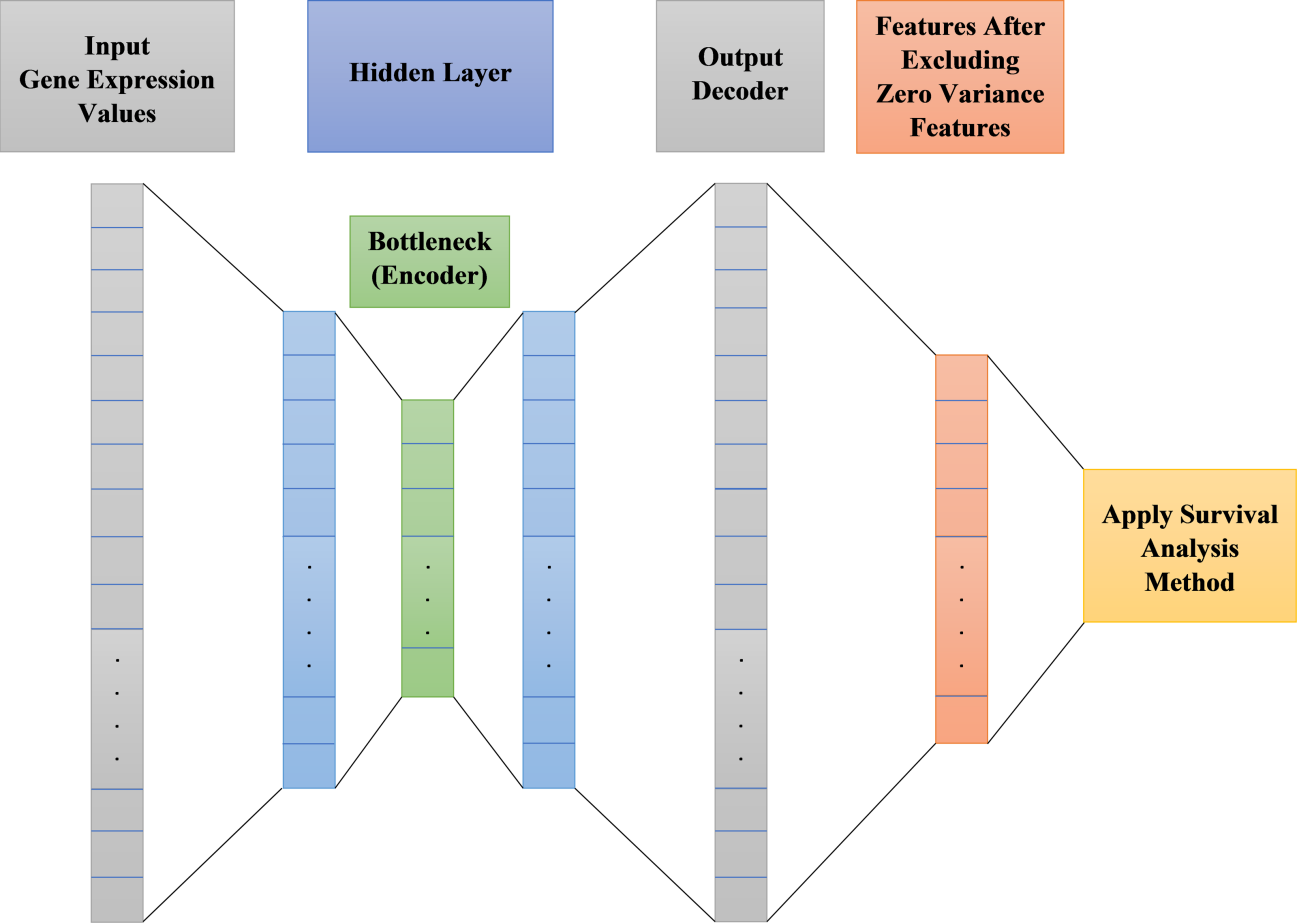
Proposed survival analysis model architecture.

Autoencoder is an unsupervised artificial neural network. It could be used to efficiently encode data then reconstructing the data back from the encoded representation and remove noise, providing a new representation of features that is as close to the original input as possible but with no noise. After noise removal from features, there will be zero variance features do not affect the output. These zero variance features are removed leaving only features that can affect the output. Figure 2 shows the main structure of our autoencoder model.

As shown in Fig. 2 the autoencoder model consists of:

• **Encoder**: This layer of autoencoder is responsible for dimensionality reduction and compressing the input data into a new encoded representation.
• **Bottleneck**: The lowest possible dimensions (compressed representation) is contained and produced in this layer.
• **Decoder**: This layer of autoencoder is responsible for data reconstruction and decoding to its original form with a new representation of the input but with less noise.
• **Loss Reconstruction**: This method is used to evaluate the decoder performance to find out how close the new decoded form to the input data.

### Autoencoder parameters tuning

The implementation of the autoencoder model on the designated datasets in their original form in this study lead to the appearance of some trammels such as high collinearity and convergence, so autoencoder deep neural network is optimized to solve these problems by reconstructing features. The number of input and output nodes for the model is dependent on the number of features in the datasets. For instance, Table 2 shows the results parameters for the autoencoder internal structure used on the first dataset shown in Table 3 (Breast Cancer Dataset (http://www.cbioportal.org/study/summary?id=brca_metabric)), after a long list of varied repeated attempts of try and error. The final model with the best results has an input layer with 24,367 nodes, three hidden layers, the first one has 3072 nodes, the second one (encoder) has 256 node and the third one has 3072 nodes, while, the output layer, which works as the decoder to reconstruct the features, has 24,367 nodes.

**Table 2:** Autoencoder model tuning parameters.

Structure	Input Layer	Hidden Layer			Dropout	Decoder (output)	Reconstruction loss	Activation function
Number of layers	1	There are three layers in the hidden layer			4	1	binary_crossentropy	Relu
Number of nodes	24367	First 3072	Hidden 256	Third 3072	Percentage (0.9)	24367	–	–

**Table 3:** Datasets description.

**Dataset**	**Target variables**		**Genes(features)**	**Training samples**	**Testing samples**
	**Classes**	**Duration**			
Breast Cancer Dataset (http://www.cbioportal.org/study/summary?id=brca_metabric)	Two Classes: death or alive	Time to the event of death or life	24367	1619	285
Hao et al. (2019)	2 classes	Dead-Alive	5567	417	105
Reddy et al. (2017)	2 classes	Dead-alive	140	801	200

In any deep learning model, the Loss functions are used to determine the error between the prediction of our model and the actual target variable. The used reconstruction loss function in this paper is *binary_crossentropy*. As for the activation function, which is used to predict the output based on the input, *Rectified Linear Unit (ReLU)* is employed in our model. ReLU can be considered as a linear function in which the input will be directly outputted if it is positive, otherwise, it will output *zero*. It has become the default activation function for many types of neural network models, because a model that uses it is easier to train and often achieves better performance. To solve the problem of overfitting, dropout layers are used. Dropout is a common and simple regularization method, which has been widely used since 2014. Simply put, dropout randomly discards some inputs during the training process. They used a neural network optimizer is Adagrad (Huang et al., 2020). Adagrad is an algorithm for gradient-based optimization. It adapts the learning rate to the parameters, performing lower learning rates for parameters associated with frequently occurring features, and higher learning rates for parameters associated with infrequent features.

### Survival analysis techniques

For high dimensional dataset, many features don’t represent the information in the data equally well. After focusing our attention on feature selection using the autoencoder, the next major stage in our model is applying the survival analysis techniques, namely cox regression and random survival forest, on the selected feature set.

#### Autoencoder with cox regression (AutoCox)

The Cox Proportional Hazards Model (Cox Regression) was first introduced by Cox (Harrell, 2001). It is a statistical method widely used in medical research to estimate various patients’ survival probability. Cox regression main idea is to account for the effect of several features at a time point and assist the relation of these features and the survival distribution. Cox is different from Multiple Regression Analysis in its dependent features is the hazard function at a given time. Its function estimation examines very small-time intervals that accommodate at most one interesting event. The parameters are estimated by maximizing the partial likelihood of the weights, where Gradient Descent algorithm is used to fit the Cox model to the data.

Cox Regression method can be considered as an example of semi-parametric models. The Cox model may be expressed with the hazard function *h(t).* ****** This hazard function gives the probability of an event happening at a given time *t* could be calculated as follows:

\begin{eqnarray*}h \left( t \right) ={h}_{0} \left( t \right) \times exp({b}_{1}{x}_{1}+{b}_{2}{x}_{2}+\cdots +{b}_{n}{x}_{n}) \end{eqnarray*}

Where: Variable *t* would be used to represent the time of survival. Determining the hazard function $h \left( t \right) $ through using n covariates (*x*_1_, *x*_2_, …, *x*_*n*_). The covariates impact would be computed by using the coefficients (*b*_1_, *b*_2_, …, *b*_*n*_). The baseline hazard can be represented by *h*_0_. The risk score of each patient is estimated as follows:

\begin{eqnarray*}R-Score={b}^{t}x \end{eqnarray*}

To calculate the values for b, the most often used method is to find the value b that maximizes cox partial likelihood function:

\begin{eqnarray*}L \left( b \right) =\prod _{s\in d}[exp(b`{x}_{r})/\sum _{i\in R}exp(b`{x}_{i})] \end{eqnarray*}

Where, *d* represents the set of the events indices (like deaths) and *R*_*r*_ is the set of indices for the patients at risk at time *t*_*r*_. In our AutoCox model implementation follows Gui & Li (2005), which proposed an iterative methodology to solve the optimization problem of estimating cox likelihood function Lasso constraints as a linear regression model.

#### Autoencoder with random survival forests (AutoRandom)

Random Survival Forest (RSF) is an ensemble learning technique is trees as the base learner (Wang & Li, 2017a). It is used to avoid the constraints of the Cox regression hazards model. The main procedure for RFS could be described as; computing a random forest using log-rank test as a splitting criterion; then calculating the cumulative hazards of each tree leaf and averaging them in for the next ensemble step. RFS trees are grown to their full size under the condition that each leaf node has no less than specific number of events.

Recently different implementations for using RFS in survival analysis have been proposed in the literature (Wang & Li, 2017a). In our AutoRandom model implementation, we focus on the most recent RFS adapted by Gui et al. (2005), where RSF procedure consists of these steps:

• Select ***S*** bootstrap samples set from the training data with size ***N*** randomly. This sample set can contain from half to two-thirds of the original number of samples, and the remaining samples are called out of bag observations.
• For each selected sample set is used to grow a survival tree based on the specified splitting criteria. For each internal node in the tree, randomly draw *candidate* covariates out of all covariates. These covariates should maximize the differentiation among the nodes or minimize the similarity risk between the nodes. Then *candidate* covariates are used for tree splitting. The stopping condition for tree growing is done when the number of samples within a leaf node is less than its current value.
• Then a cumulative hazard function particular leaf node at time ***t*** for each tree is estimated using this equation:
  \begin{eqnarray*}{F}_{h}(t)=\sum _{{t}_{l,h}\lt t} \frac{{d}_{l,h}}{{p}_{l,h}} \end{eqnarray*}

Where *d*_*l*,*h*_ is the number of specific evet (i.e., death) at risk at time *t*_*l*,*h*_, and *p*_*l*,*h*_ is the numbers of patients at risk at time *t*_*l*,*h*_.

## Results

### Dataset

The microarray dataset is organized as just an array. The functions (genes) are expressed by array rows whereas the columns are used to represent the instances. The microarray comprises two arrays: one for data from instruction, and the other for data processing. Here, the autoencoder is applied to the training data array, resulting in a new subset of data with less noise. There will be some zero-variance features as a result of applying autoencoder, these features are removed as they do not affect our system. The reduced subset of trains will be used to train the proposed framework. Testing data array is used as an evaluation for the proposed framework. All information about the used dataset is shown in Table 3. Three datasets are used to evaluate our model. The first dataset is Breast Cancer (http://www.cbioportal.org/study/summary?id=brca_metabric) (METABRIC, Nature 2012, and Nat Commun 2016) dataset, having 24,367 features and 1,904 samples, 1,103 of the samples have been dead and the other 801 are still alive. The second dataset is one of the datasets used in an existing method called COX-PASnet (Hao et al., 2019). This dataset has 522 samples and 5,567 features with 75 sample alive and 447 dead. While the third dataset is downloaded from Cancer Genome Atlas (TCGA, http://cancergenome.nih.gov), and contains 140 features and 1001 samples for Diffuse Large B Cell Lymphoma (DLBCL) tumors.

For evaluating our model on this dataset, a personal computer that has the following specifications is utilized: 8 GB Random Access Memory (RAM), core i7, 8^*th*^ generation and 4 GB Nvidia GPU (GTX 1650).

### Evaluation parameters

To evaluate our proposed models, concordance index (Raykar, Steck & Krishnapuram, 2007) and Kaplan–Meier Estimate (Heller & Mo, 2016) are used. The concordance index is similar to measuring accuracy in classification problems but case survival analysis. Concordance is used to indicate how well the survival model is. Concordance index ranges from 0 to 1. If concordance equals zero, this means perfect anti-concordance. If concordance equals one, this means a perfect model. If concordance is greater than or equal to 0.7, this means a good model. If concordance is greater than or equal to 0.8, this means a strong model. The mathematical expression for concordance index can be represented by equation 2 (Raykar, Steck & Krishnapuram, 2007):

\begin{eqnarray*}\text{Co-index}=  \frac{1}{{|}\varepsilon {|}} \sum _{{\varepsilon }_{i j}}{1}_{g({t}_{i}) \lt g({t}_{j})} \end{eqnarray*}

Where, the number of edges in the order graph can be represented by $ \left\vert \varepsilon \right\vert $, and the predicted survival time for an item *i* is represented by $g \left( {t}_{i} \right) $.

Kaplan–Meier Estimate (KME) is used to measure accurately the percentage of samples in the dataset in which the patient survives after a particular amount of time. KME is a simple and effective method to estimate the survival rates; where, for two or more sample groups, a log-rank test is calculated to measure the difference among their survival distributions. The difference between survival outcomes for the two groups is considered significant if its log-rank test *P*-value less than 0.05. In this paper, we have only two groups of samples (death and alive) therefore, KME *P*-value between the two groups is estimated and reported as performance evaluation parameter.

### Survival analysis with/without feature selection

We compare our proposed autoencoder feature selection technique for survival analysis with the widely used feature selection algorithm, Principal Component Analysis (PCA) (Torkey et al., 2020) and Feature Importance (FI) (Wang & Li, 2017b). Then using these selected feature sets for survival analysis and computing Co-index and *p*-value for KME. Table 4 shows a comparison for p-vale and Co-index among four models in four cases. The first case is when not using any feature selection techniques. While, the second case is using PCA for feature reduction. Here PCA is used to reduce the features and test with random forest classifier to get the most significant feature set for classifying the data based on two classes death and alive, where PCA reduced all features into a set of 56 features. The other two cases are with using FI algorithm developed in (Salem, Attiya & El-Fishawy, 2016), and finally our proposed models.

**Table 4:** Evaluation results for survival analysis in the case of ML feature selection algorithms, using our proposed model and NOT using feature selection at all. Comparison of proposed system and previous studies in Terms of Concordance Index.

**Dataset**	**Feature selection methods**	**Cox Regression**		**Random Survival Forest (RSF)**	
		***P*-value**	**Co-index**	***P*-value**	**Co-index**
Breast Cancer Dataset (http://www.cbioportal.org/study/summary?id=brca_metabric)	Without feature selection	–	–	0.037	0.59
	PCA feature reduction	0.073	0.62	0.061	0.64
	Feature Importance (FI)	0.0490	0.67	0.0290	0.62
	Proposed AutoEnconder model	**0.051**	**0.77**	**0.042**	**0.92**
Hao et al. (2019)	Without feature selection	0.0542	0.62	0.0756	0.67
	PCA feature reduction	0.0705	0.70	0.0521	0.72
	Feature Importance (FI)	**0.0490**	**0.67**	0.0910	0.65
	Proposed AutoEnconder model	0.051	0.63	**0.082**	**0.73**
Reddy et al. (2017)	Without feature selection	0.058	0.58	0.079	0.76
	PCA feature reduction	0.089	0.97	0.081	**0.98**
	Feature Importance (FI)	0.0490	**0.91**	0.0290	**0.99**
	Proposed AutoEnconder model	**0.010**	0.61	**0.052**	**0.98**

**Note.**

Values in bold represent the best results for each dataset when using different feature selection algorithms.

**Figure 3:**
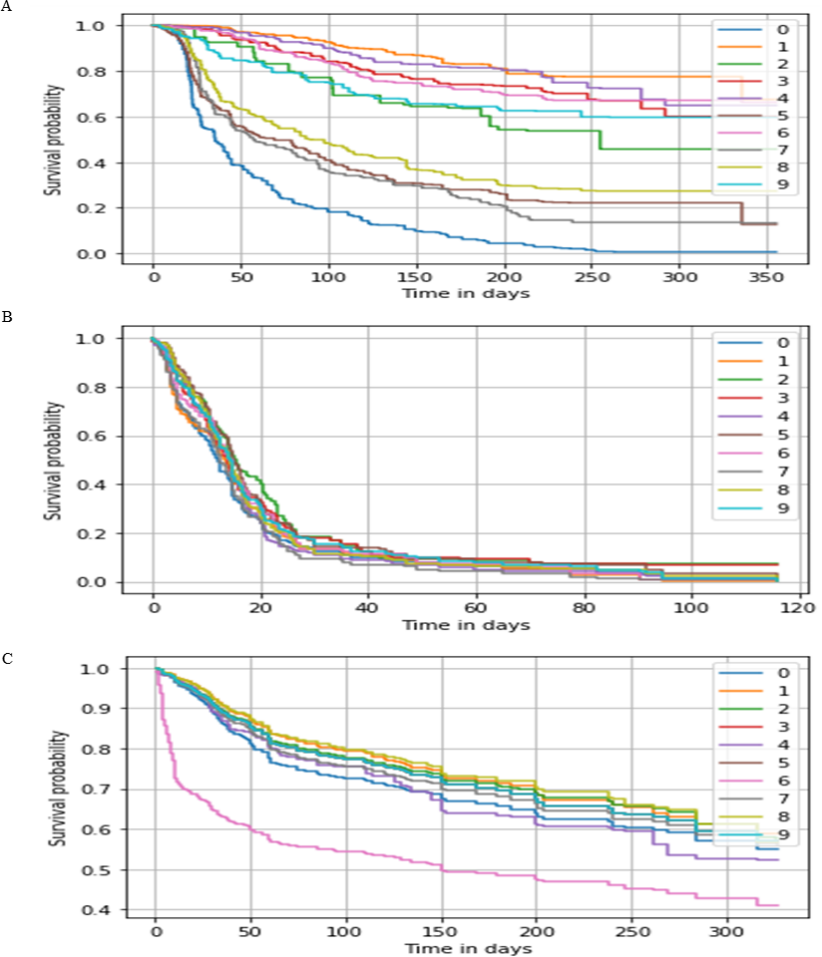
Survival probability prediction using AutoRandom in each dataset. Survival probability prediction for random 10 patients (A) represents the probability of survival for a random 10 patients for the breast cancer dataset, while (B) Survival probability prediction in Hao et al. (2019) dataset, and (C) Survival probability prediction for Reddy et al. (2017) dataset.

As shown in Table 4 using the whole feature set of 24, 367, cox regression was not able to provide any result in visible time after a considerable number of trials, while RSF co-index was equal to 0.59, 0.67, and 0.76 with 0.037, 0.075, and 0.079 KME *p*-value for the three datasets respectively. It is found from this results the when the number of feature reduced to the range of couples of thousand cox regression was able to provide results in a resendable time frame. On the other hand, when using techniques such as PCA and FI for reducing the features leads to improve the performance by a range of 5.3 for Co-index with an acceptable *p*-value.

As for our proposed model AutoCox which has a training concordance of 0.79, 0.75, and 0.99 and a testing concordance of 0.77, 0.73, 0.98 consider to be a good performance comparing to the other models. AutoRandom has a training concordance of 0.93 and a testing concordance of 0.921 which means that it is an excellent model. Figure 3 shows a sample of the predicted survival probability for 10 patients of the testing data using AutoRandom, as the best performing model from the result analysis on the three datasets. Based on our observations from the results, the number of input feature of the data greatly affect the performance of the model. Other feature selection/reduction techniques influence by the survival analysis method used. Also, RSF algorithm provides better results whih most reduction techniques.

As applying any deep learning model on many features requires a significant amount of time, our model was developed to run on GPU that lead to a remarkable reduction in the model training time. Table 5 shows the performance of the proposed models with and without GPU and computing survival analysis without applying the autoencoder neural network. For instance, on the first dataset AutoCox without GPU, it took about 10.67 h which can be considered a long running time. For reducing the run time, the autoencoder was applied on the GPU resulting run time that is up to 0.365 h. While other two datasets with fewer number of features required at most less than 2 h for estimating the AuroCox results without using GPU and less than 2 min with GPU. As for AutoRandom, it is found to be much faster specially when the number of features is around one hundred as in the third dataset.

**Table 5:** Run-time comparison between different survival systems.

Methods	Random survival without deep learning	AutoCox without GPU	AutoCox with GPU	AutoRandom without GPU	AutoRandom with GPU
Breast cancer dataset (http://www.cbioportal.org/study/summary?id=brca_metabric)	0.161 h	12.65 h	0.563 h	10.676 h	0.373 h
Hao et al. (2019)	1.33 h	1.52 h	1.33 min	42.3 min	12.1 s
Reddy et al. (2017)	34.5 min	32.6 min	0.83 min	10.6 min	10.4 s

The results show that applying autoencoder deep neural network before applying survival analysis methods would solve problems. It can solve problems whether by speeding up in some cases (when applied on GPU), increasing the prediction accuracy (random survival forests) or in some cases it provides the possibility to apply some survival analysis methods on a large number of features.

### Comparison with the existing deep learning models

**Figure 4:**
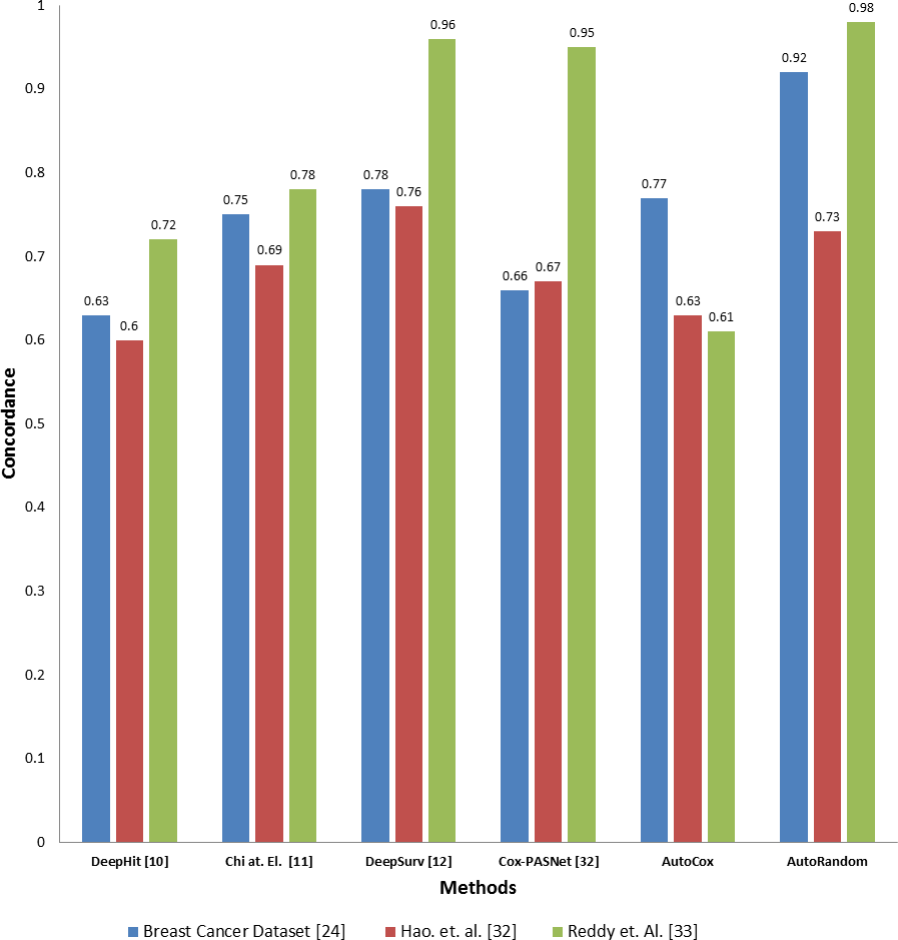
Comparison between the proposed models and four of the previous methods in terms of concordance index when applying to the three datasets.

To evaluate the effectiveness of our proposed model compared to the existing deep learning base survival analysis approaches, we download, run and evaluate four of the most recent and known deep models, including DeepHit, DeepSev, deep survival analysis models proposed by Chi, Street & Wolberg (2007) and Cox-PASNet (Hao et al., 2019). A comparison between the two proposed models concordance index value and other deep learning models applied on the same datasets is presented in Fig. 4. Employing our feature selection deep autoencoder model improve the performance over the existing model. As the model shows a significant increase in the Co-index (up to 0.98 comparint to 0.92) in the case of using RSF over the other models, while DeepHit and AutoCox were slightly inan equal range with 0.752 and 0.77 respectively in some cases of the dataset. Concordance indix clearly shows different distribution with different features set input to each model. In some cases, Autocox share the close index value with the existing model. However, our autoencoder model is based on removing the noise from the data, which here represents the unrelated features to the problem of survival estimation. Also, our models trained on many samples to avoid the problem of overfitting and tested on a completely unseen before test data sunset.

### Selection of genes related to survival (biomarkers)

For each gene resulting from the autoencoder, the *p*-value, and coefficient are computed. The *p*-value is used to show the degree of effect of each gene upon the survival probability. If the *P*-value for a feature is less than 0.05, this means that it has a big impact on the survival probability, and any changes in the predictor’s value; changes in the response variable would be made. A positive coefficient would indicate worse prognosis and a negative coefficient would indicate a protective effect of the variable with which it is associated. *P*-value which is considered as an indicator for the extent of influence of the most important features on the survival probability and coefficient is contained in Table 6. For each feature, the *p*-value is computed for testing the null hypothesis that the coefficient is equal to zero (no effect). For a predictor, if *P*-value is lower than 0.05 this means that this predictor has a major effect on survival probability and changes in this predictor would affect the survival probability. A worse prognosis can be indicated by a positive coefficient and a protective effect of the variable with which it is associated can be indicated by a negative coefficient.

**Table 6:** AutoCAD and AutoRandom models combined the most important Genes Coefficient and *P*-Value.

**Breast cancer dataset (http://www.cbioportal.org/study/summary?id=brca_metabric) dataset**			**Hao et al. (2019) dataset**			**Reddy et al. (2017)**		
Gene (Feature)	*P*-value	Coefficient	Gene (Feature)	*P*-value	Coefficient	Gene (Feature)	*P*-value	Coefficient
BU739243	0.045	0.11	CARD11	0.035	−0.12	AANAT	0.348	0.13
S100P	0.004	0.12	CD22	0.118	−0.11	ABCB8	0.442	0.17
AI769787	0.045	0.1	CHD1	0.196	0.21	ABCD1	0.487	0.15
AI056267	0.047	0.07	CIITA	0.104	0.47	ACO1	0.486	−0.16
TTLL7	0.025	0.115	DCAF6	0.002	0.155	ALAS1	0.498	−0.95
SUN2	0.029	−0.106	EBF1	0.053	−0.19	ALDH3A2	0.415	−0.11
MEST	0.015	−0.095	ETS1	0.050	0.175	AMPD1	0.487	−0.142
CD74	0.02	−0.11	EZH2	0.134	−0.251	AOC3	0.383	−0.11
BM888205	0.014	−0.12	FAM5C	0.176	−0.142	APC	0.473	0.21
RAC1	0.012	−0.11	IL16	0.117	0.311	ARHGAP24	0.467	0.67
PTPRG	0.045	0.11	IRF4	0.040	0.21	ATF3	0.479	0.155
BG683264	0.004	0.12	MSH6	0.055	0.167	ATP5G2	0.362	−0.166
AK094562	0.045	0.1	NFKBIE	0.099	0.155	ATP6V0B	0.447	−0.75
MYST1	0.047	0.07	POU2F2	0.088	−0.16	AUH	0.498	−0.51
AW297374	0.025	0.115	PTPN6	0.162	0.15			
DB299637	0.029	−0.106	TBL1XR1	0.071	−0.151			
AA613795	0.015	−0.095						
ZBTB8B	0.02	−0.11						
CSNK1A1	0.014	−0.12						
CD84	0.012	−0.11						

Table 6 shows AutoCox and AutoRandom models combined most important Genes Coefficient and P-Value. For instance, in breast cancer dataset, some of these genes were discovered experimentally to be considered as gene biomarkers for survival probability, like, PTPRG, MYST1, BG683264, and AK094562, S100P, AI056267, TTLL7, SUN2, CD74, BM888205 and RAC1.

## Discussion

Survival analysis is a model for analyzing the expected duration of time until an event could happen. This problem encounters several challenges such as the number of features used in the analysis and difficulty in acquiring sufficient samples. There are many current approaches for conducting this sort of survival study. most of these approach are based on regression models that is commonly used in survival analysis. In this study, we developed two approaches using a powerful deep learning autoencoder model, AutoCox and AutoRandom. The results show that using deep learning to reduce and select a specific set of genes, which play major roles in defining the survival probability of the patients, can enhance the accuracy of a powerful estimation. Comparing to using other feature reduction methods like PCA and FI our autoencoder model provides better selection for the related features, as the results show huge differences between results generated using our model and other methods. The proposed model can facilitate better approach to predict the related genes (features in our datasets) to the patient survival and hence assist in diagnosis and treatment of different disease.

**Figure 5:**
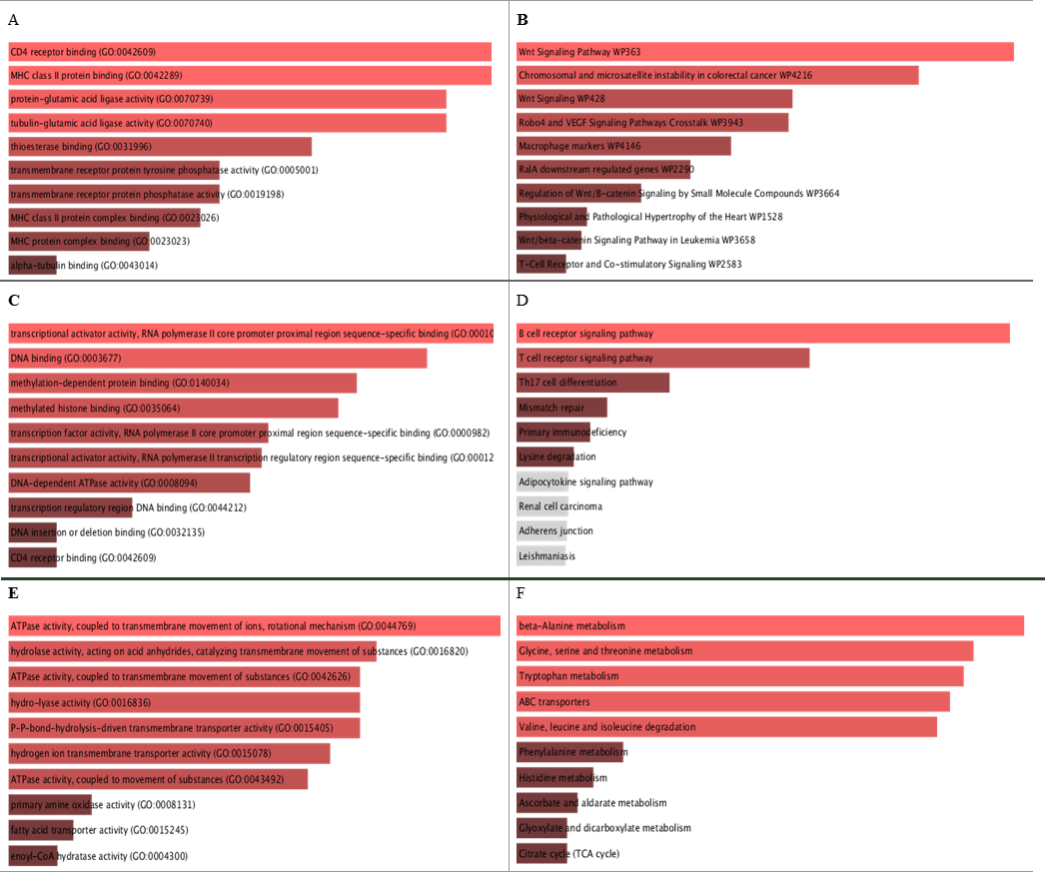
GO molecular function and KEGG pathways analysis for the biomarker results from the three datasets. A, C, and E are the bar graphs that show the top 10 enriched molecular functions for the selected biomarkers, and B, D, and F represent the top 10 enriched KEGG pathways for the three datasets Breast Cancer Dataset (http://www.cbioportal.org/study/summary?id=brca_metabric), dataset Hao et al. (2019), and dataset Reddy et al. (2017) repectively.

**Figure 6:**
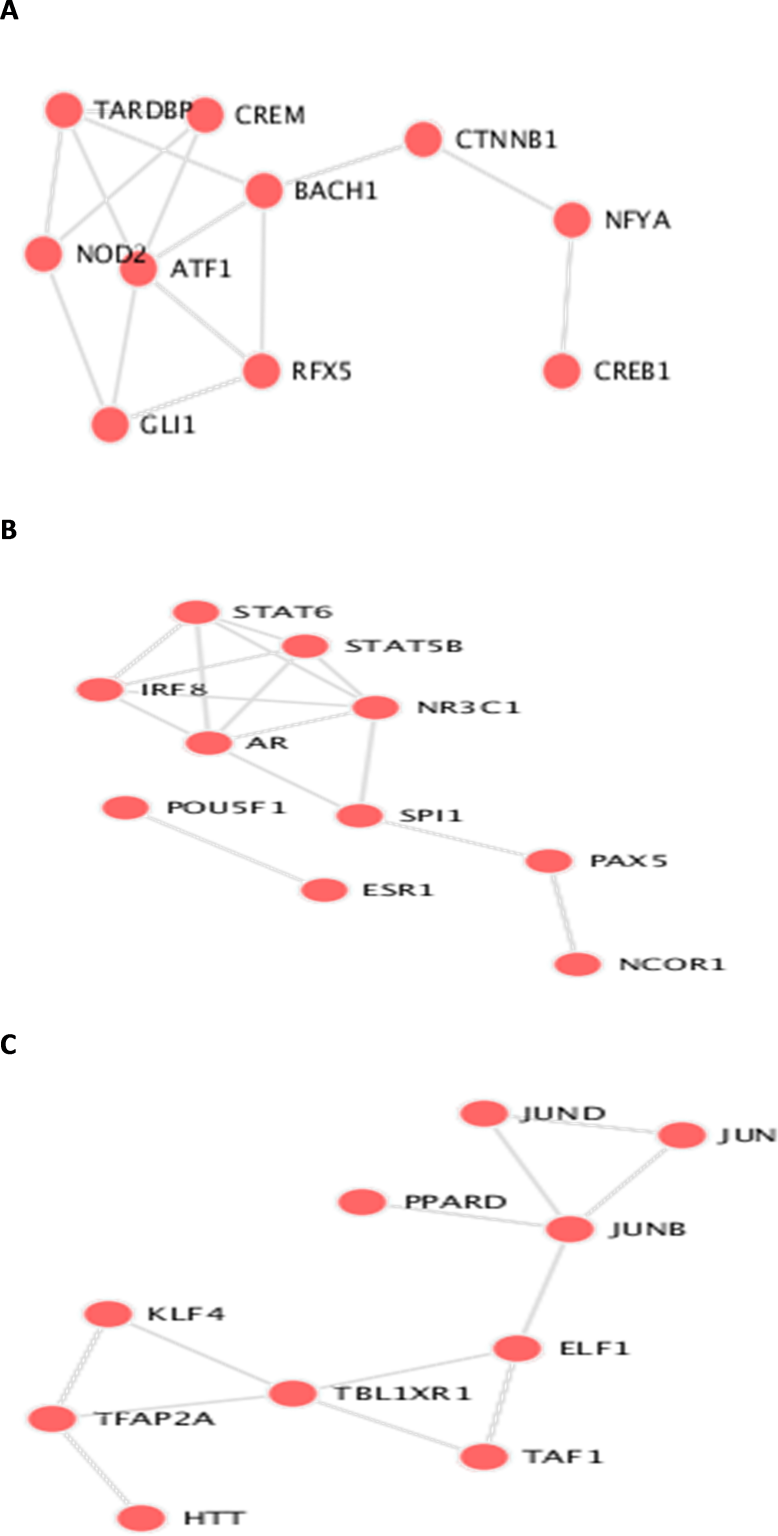
Transcription Factor protein-protein interactions (PPIs) network for each biomarker set, A, B, and C are the PPIs for the interacting transcription factor genes of the the three datasets.

### Biological pathways and GO molecular function enrichment analysis

The identifieed biomarker for each dataset is analyzed using their enriched GO molecular functions and biological pathways using EnrichR R package version 2.0 (Kuleshov et al., 2016), where GO terms and biological function were selected from 2019 databases and KEGG pathways from 2019 databases. In this analysis, GO functional process and KEGG pathways are considered signi?cant if their adjusted *p*-value is less than 0.05. EnrichR is used to plot the bar diagram for showing the most enriched GO function and pathways as shown in Fig. 5. The tables for the full biological processes and pathways analysis along with the adjusted *P*-value and the Combined Score are presented in supplementary martials. For the first dataset, KEGG pathway analysis revealed that the discovered biomarkers are highly associated with pathways including “Wnt signaling pathway WP363” and “Robo4 and VEGF signaling Crosstalk WP3943” which are known to play a role in cancer progression (Sever & Brugge, 2015). Transcription factors are proteins that control the rate of mRNA transcription that can regulate genes expression. Figure 6 shows the protein-protein interactions (PPIs) network for the interactions among the transcription factors regulating these biomarkers discovered in each dataset. From the figure, we can see that these factors are highly interacting, which shown these biomarkers are regulated by hub genes and are some of major complexes.

## Conclusion

In this study, we proposed two deep learning-based survival prediction models, AutoCox and AutoRandom. This can help patients estimating risks, guiding their options for treatment, and avoiding ineffective treatments. They are a combination of a deep autoencoder neural network and hazard function-based survival analysis techniques, in order to deal with datasets with thousands of features. Our model is evaluated on RNAseq Breast Cancer microarray dataset. The main contribution of our models is enhancing the survival probability estimation measured on concordance index, as shown by increasing the concordance index values by about 13.9 using AutoRandom. Moreover, the proposed autoencoder was mainly used to select and rank the input features based on the target events on the dataset. That enables our model to discover the most related features/genes for survival. Implementing out models on GPU was able to speed up the process especially for cox regression on data with a lot of features, as results show that AutoCox applied on GPU requires about 1.5 h, and without using GPU about 11.41 h, while applying cox regression without using deep learning resulted in resources exhausted and didn’t work.

This work built the models and evaluated their performance by exclusive training and testing then on a single dataset with only two events. More datasets with multiple events may improve the results and further validate the benefits of deep autoencoder-based survival analysis prediction. Furthermore, incorporating more omics data, such as clinical data, DNA methylation and mutation considered to be crucial in describing human complex diseases. A model that could integrate multi-omics data is one of our desirable directions for future work.

## Additional Information and Declarations

### Data Availability

The following information was supplied regarding data availability: Data and Code are available at GitHub: https://github.com/Mostafa-Samy-Atlam/Autoencoder-with-survival-analysis-methods.
